# A Novel Intracellular Isoform of Matrix Metalloproteinase-2 Induced by Oxidative Stress Activates Innate Immunity

**DOI:** 10.1371/journal.pone.0034177

**Published:** 2012-04-03

**Authors:** David H. Lovett, Rajeev Mahimkar, Robert L. Raffai, Leslie Cape, Elena Maklashina, Gary Cecchini, Joel S. Karliner

**Affiliations:** 1 Department of Medicine, San Francisco Department of Veterans Affairs Medical Center, University of California San Francisco, San Francisco, California, United States of America; 2 Department of Surgery, San Francisco Department of Veterans Affairs Medical Center, University of California San Francisco, San Francisco, California, United States of America; 3 Department of Biochemistry and Biophysics, San Francisco Department of Veterans Affairs Medical Center, University of California San Francisco, San Francisco, California, United States of America; 4 The Cardiovascular Research Institute, University of California San Francisco, San Francisco, California, United States of America; National Institutes of Health, United States of America

## Abstract

**Background:**

Experimental and clinical evidence has pinpointed a critical role for matrix metalloproteinase-2 (MMP-2) in ischemic ventricular remodeling and systolic heart failure. Prior studies have demonstrated that transgenic expression of the full-length, 68 kDa, secreted form of MMP-2 induces severe systolic failure. These mice also had unexpected and severe mitochondrial structural abnormalities and dysfunction. We hypothesized that an additional intracellular isoform of MMP-2, which affects mitochondrial function is induced under conditions of systolic failure-associated oxidative stress.

**Methodology and Principal Findings:**

Western blots of cardiac mitochondria from the full length MMP-2 transgenics, ageing mice and a model of accelerated atherogenesis revealed a smaller 65 kDa MMP-2 isoform. Cultured cardiomyoblasts subjected to transient oxidative stress generated the 65 kDa MMP-2 isoform. The 65 kDa MMP-2 isoform was also induced by hypoxic culture of cardiomyoblasts. Genomic database analysis of the MMP-2 gene mapped transcriptional start sites and RNA transcripts induced by hypoxia or epigenetic modifiers within the first intron of the MMP-2 gene. Translation of these transcripts yields a 65 kDa N-terminal truncated isoform beginning at M^77^, thereby deleting the signal sequence and inhibitory prodomain. Cellular trafficking studies demonstrated that the 65 kDa MMP-2 isoform is not secreted and is present in cytosolic and mitochondrial fractions, while the full length 68 kDa isoform was found only in the extracellular space. Expression of the 65 kDa MMP-2 isoform induced mitochondrial-nuclear stress signaling with activation of the pro-inflammatory NF-κB, NFAT and IRF transcriptional pathways. By microarray, the 65 kDa MMP-2 induces an innate immunity transcriptome, including viral stress response genes, innate immunity transcription factor IRF7, chemokines and pro-apoptosis genes.

**Conclusion:**

A novel N-terminal truncated intracellular isoform of MMP-2 is induced by oxidative stress. This isoform initiates a primary innate immune response that may contribute to progressive cardiac dysfunction in the setting of ischemia and systolic failure.

## Introduction

Matrix metalloproteinases play a central role in many forms of cardiovascular disease, including valvular disease, ischemia/reperfusion injury, compensatory hypertrophy, post-infarction remodeling and systolic heart failure (Reviewed in [1], [2]). The human matrix metalloproteinase (MMP) gene family is comprised of multiple members with a remarkable diversity of structure, function and regulation. The current nomenclature of this gene family is based on the preferred extracellular matrix molecules cleaved by each enzyme. The gene family has been divided into subgroups consisting of interstitial collagenases (MMP-1,-8,-13), the stromelysins (MMP-3, -10, -11), the matrilysins (MMP-7,-26), the membrane-type MMPs (MT-MMP1-6) and the gelatinases (MMP-2, -9). The proteins share several distinguishing features, included a conserved modular structure, secretion in an inactive zymogen form and dependence on zinc for catalytic activity.

From this diverse group it has become increasingly evident that a specific metalloproteinase, MMP-2, is of central pathophysiologic and therapeutic importance in cardiovascular disease [1], [2]. The primary mRNA transcript for MMP-2 encodes a protein with an apparent molecular mass of 68 kDA consisting of a short N-terminal signal sequence for ER/Golgi/secretory vesicle processing, a propeptide domain which maintains enzymatic latency, and a highly conserved zinc-binding catalytic domain in conjuction with hemopexin and fibronectin domains important for binding to extracellular matrix substrates. Enzymatic latency is maintained by the “cysteine switch” mechanism, in which a cysteine residue in the prodomain sequence PR**C**GVN is folded over the zinc-containing catalytic site. Classical proteolytic activation in the extracellular space is a complex process resulting from the interaction of the latent MMP-2 with MT1-MMP/TIMP2 complexes on the cellular surface, resulting in cleavage of the prodomain and acquisition of enzymatic activity [3], [4].

Until recently, nearly all studies had focused on the extracellular actions of MMP-2 in the genesis of cardiac dysfunction. This appeared reasonable in view of the above described structure of the molecule, with the typical features of a secreted protein. Coker, et al. [5] provided the first evidence for the existence of intracellular MMP-2. Isolated left ventricular cardiomyocyte preparations were cultured for short periods and evaluated for MMP-2 secretion. Surprisingly, immunofluorescence staining of the isolated cardiomyocytes revealed prominent MMP-2 staining within the myocyte in a pattern consistent with both sarcomeric and sarcolemmal localization. Subsequently, the Schulz laboratory published a pivotal paper showing physical association of latent, full-length 68 kDa MMP-2 with the cardiac sarcomere [6]. A direct interaction with sarcomeric troponin I was demonstrated and the authors provided evidence for MMP-2-mediated troponin I degradation following acute ischemia/reperfusion (I/R) injury. As reported, [7], [8], [9], activation of latent full length 68 kDa MMP-2 in the setting of I/R injury is mediated by the nonproteolytic action of reactive oxygen and nitrogen species to open the cysteine switch.

We have reported on the phenotype of cardiac-specific transgenic mice expressing a constitutively active, full-length (68 kDa) isoform [10]. Our initial hypothesis was that cardiac expression of active MMP-2 would primarily affect the structure and composition of the cardiac extracellular matrix. While there was a significant increase in cardiac extracellular matrix in these mice, the most remarkable phenotypic findings related to contractile abnormalities and severe systolic dysfunction. In addition, ultrastructural studies demonstrated cardiomyocyte myofilament lysis and mitochondrial structural abnormalities. Furthermore, these mice did not respond to ischemic preconditioning in isolated heart preparations and exhibited unanticipated abnormalities in mitochondrial morphology, mitochondrial respiration, lipid peroxidation and recovery of contractile function following ex vivo ischemia/reperfusion injury [11]. These findings suggested that specific mitochondrial abnormalities were induced by MMP-2 expression. Given these observations, we hypothesized that a second intracellular MMP-2 isoform may exist which affects cardiomyocyte structure and function, in part through interactions with mitochondria. In this report we provide a detailed characterization of a novel intracellular, N-terminal truncated 65 kDa isoform of MMP-2 generated by oxidant stress which activates a pro-inflammatory, pro-apoptotic innate immune response.

## Results

### Detection of a Mitochondrial-Associated 65 kDa MMP-2 Isoform in Three Murine Models of Cardiac Injury

**Figure 1:**
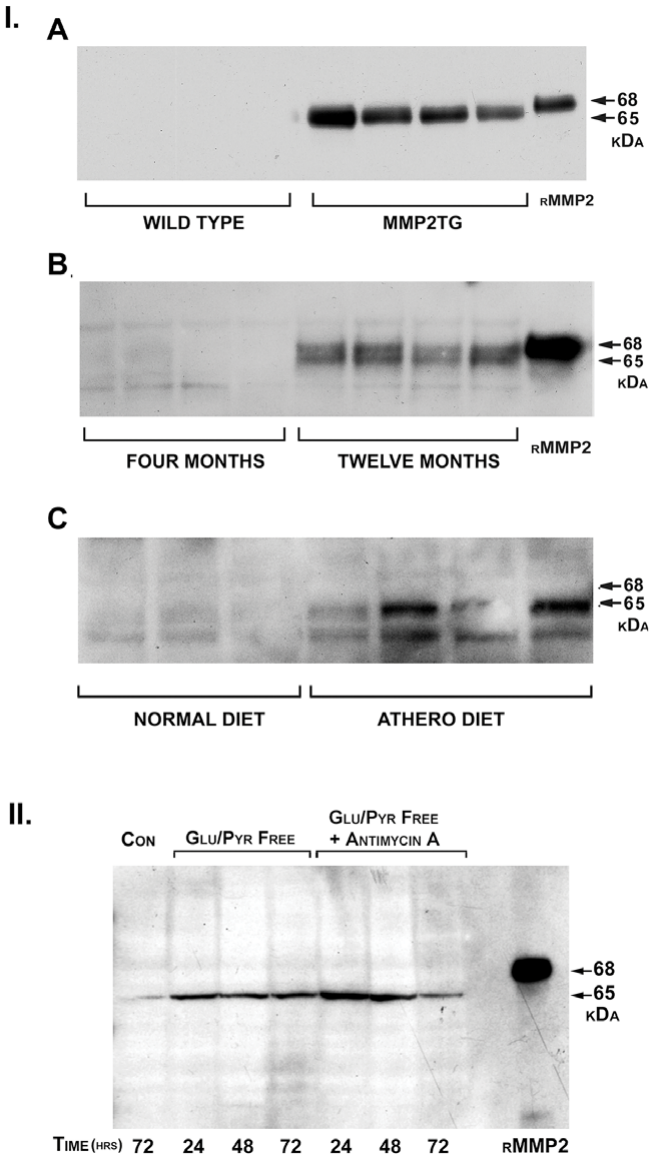
Detection of a truncated MMP-2 isoform in mitochondrial-enriched fractions from murine hearts and cardiomyoblast H9C2 cells. I. A. Western blot analysis for MMP-2 expression in mitochondrial-enriched fractions from left ventricles of four and twelve month old wild type CD-1 mice (n = 4 for each group). MMP-2 bands with apparent molecular masses of 65 kDa are detected in the mitochondrial fractions from the twelve month old mice, but not in the fractions from four month old mice. (rMMP-2: recombinant full-length 68 kDa MMP-2 protein). I. B. Western blot analysis of mitochondrial-enriched fractions from left ventricles of hypomorphic SR-BI KO/ApoER61^h/h^ mice fed a normal diet or a high fat atherogenic diet for 30 days (n = 3–4). MMP-2 bands of 65 kDa and 62 kDa are detected in the mitochondrial fractions of mice fed an atherogenic diet. (Figure S2 shows a mitochondrial fraction run in parallel with recombinant 68 kDa MMP-2). II. *In vitro* model of transient inhibition of oxidative phosphorylation (OxPhosI). Partial OxPhosI was induced by incubation for 15 minutes in mitochondrial substrate glucose/pyruvate-free DMEM as detailed in Methods, followed by restoration in complete medium. More complete OxPhosI was induced by inclusion of antimycin A (2 µM) and 2-deoxyglucose (10 mM) in substrate-free medium. Westerns blots of mitochondrial-enriched fractions were performed at 24, 48 and 72 hours following OxPhosI. The 65 kDa MMP-2 isoform was detected in the mitochondria-enriched fractions from the H9C2 cells subjected to partial inhibition of OxPhosI and this was increased in the fractions from cells subjected to more complete OxPhosI with antimycin A and 2-deoxyglucose. (rMMP2: recombinant full-length 68 kDa MMP-2).

We recently reported that hearts from mice expressing a constitutively active full-length 68 kDa MMP-2 transgene exhibited a number of abnormalities, including systolic dysfunction and fibrosis, coupled with mitochondrial structural and functional defects [10], [11]. Interestingly, as these mice aged and developed systolic dysfunction there was a large increase in endogenous (i.e. non-transgene) MMP-2 expression [10]. Western blots of isolated cardiac mitochondria detected a 65 kDa MMP-2 protein not derived from the epitope-tagged MMP-2 transgene and not present in age-matched litter mate controls (Figure 1, I., A). These observations generated the hypothesis that a previously undetected intracellular MMP-2 isoform induced within the context of the failing ventricle could contribute to the observed mitochondrial defects.

To exclude the possibility that artifacts arising from aberrant MMP-2 transgene expression or transgene transcript processing were responsible for the observed 65 kDa MMP-2 protein, we performed Western blots of cardiac mitochondria-enriched preparations from ageing mice. We also examined cardiac mitochondrial preparations from a murine model of cardiovascular disease within a non-MMP-2 transgenic context. The mitochondrial isolation procedures used generate highly enriched preparations of mitochondria with minimal cytoplasmic or endoplasmic reticulum contamination (Figure S1). As an important technical note, (see Methods), the 65 kDa MMP-2 isoform was present in very limited amounts (∼1–5 ng/200 µg mitochondrial lysate). We used an affinity capture technique to exploit the selective binding of the fibronectin-like domains of the MMP-2 protein to immobilized gelatin [10]. Due to the large MMP-2 binding capacity of immobilized gelatin, this technique quantitatively captures all ambient MMP-2 protein present in cellular fractions. Using this approach it is not possible to use conventional Western blot protein loading controls.

Figure 1, panel I., B, shows a representative Western blot for MMP-2 of mitochondria-enriched preparations isolated from 4 and 12 month old wild type CD-1 mice. No MMP-2 signal is present in the 4 month mitochondria preparations, while a clear signal is present in the mitochondria preparations from 12 month old mice. The apparent molecular mass of the MMP-2 band in the mitochondria preparations is 65 kDa and clearly migrates faster than purified recombinant MMP-2, which has an apparent molecular mass of 68 kDa. We also examined mitochondria-enriched preparations from hypomorphic ApoER61^h/h^/SR-BI KO mice. These mice express very low levels of ApoE, and coupled with the knockout of the SR-B1 scavenger receptor, are a reproducible model of accelerated coronary atherogenesis and myocardial infarction when placed on a high fat diet for thirty days [12]. While a 65 kDa MMP-2 isoform was not detected in the mitochondria-enriched fractions from mice maintained on a normal diet, (Figure 1, Panel I., C), it was readily detected, along with a less abundant 62 kDa isoform, in the mitochondria-enriched fractions of mice maintained for 30 days on the atherogenic diet. We did not detect MMP-2 bands of apparent molecular mass of 68 kDa (i.e. full length MMP-2) in these mitochondrial preparations. (Figure S2 shows a representative mitochondrial fraction from these mice showing a 65 kDa MMP-2 band run in parallel with the recombinant 68 kDa full length MMP-2 control.)

### Transient Oxidative Stress Induces the 65 kDa MMP-2 Isoform *in Vitro*

Both normal ageing and myocardial infarction are associated with increased oxidative stress [13], [14]. This suggested to us that the truncated 65 kDa isoform of MMP-2 was possibly generated as a consequence of reactive oxygen species production. Therefore, we established an *in vitro* cellular model using cardiomyoblast H9C2 cells to determine the potential effects of transient oxidative stress on formation of the 65 kDa MMP-2 isoform. We established a two-step method to induce graded degrees of mitochondrial stress generated by transient inhibition of oxidative phosphorylation (OxPhosI) [15]. Lower levels of OxPhosI were induced by incubating cells in DMEM lacking D-glucose and sodium pyruvate for 15 minutes, thereby depriving the cells of substrates required for mitochondrial respiration. Higher levels of OxPhosI were induced by including the mitochondrial Complex III inhibitor antimycin A (2 µM) and 2-deoxyglucose (5 mM) in the medium for 15 minutes. Thereafter, the cells were washed and cultured in complete medium for 24 to 72 hours. Western blots for MMP-2 were performed on mitochondria-enriched fractions.

As shown in Figure 1, panel II., incubation in mitochondrial respiration substrate-free medium for 15 minutes followed by return to complete medium was sufficient to induce synthesis of the 65 kDa MMP-2 isoform, which was readily detected at 24 hours and unchanged by 72 hours. Addition of the Complex III inhibitor antimycin A, in combination with 2-deoxyglucose, increased the levels of the mitochondria-associated 65 kDa MMP-2 isoform. Thus, a transient period (15 min) of graded inhibition of mitochondrial respiration, with subsequent ROS generation, leads to graded increases in synthesis of a 65 kDa MMP-2 isoform associated with mitochondria.

### Mechanism of 65 kDa MMP-2 Isoform Generation by Hypoxia

Protein sequence diversity arising from an individual gene is most frequently accomplished by means of alternative transcript splicing [16]. As detailed in the Discussion section, there is no genomic database evidence for alternative MMP-2 transcript splicing as a mechanism for the generation of a 65 kDa isoform.

**Figure 2:**
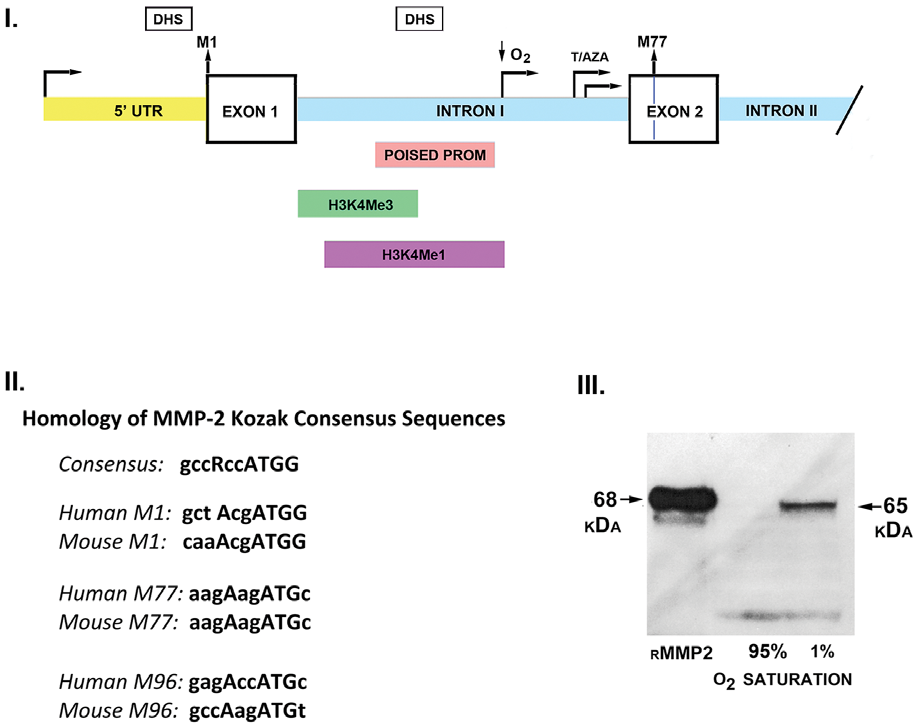
Database-mapped alternate transcriptional start sites in first intron of MMP-2 gene-activation by hypoxia. I. Schematic diagram of the MMP-2 gene. The full length 68 kDa protein is encoded by a transcript generated by the canonical transcriptional start site (TSS) located in the 5′ flanking region of the MMP-2 gene. M^1^ is located within the first exon. Mapped alternate TSS’s are located in the 3′ end of the first intron and are induced by hypoxia or epigenetic stress (arrows). Transcripts generated from these TSS encode a 65 kDa MMP-2 protein beginning at M^77^ located within the second exon. Boxes below intron I denote chromatin structures characteristic of a poised promoter and histone marks characteristic of promoter (H3K4me3) and enhancer (H3K4me1) elements from the ENCODE project. Solid boxes above the gene sequence denote mapped DNAse hypersensitivity (DHS) sites. II. The N-terminus of the MMP-2 gene contains three in-frame Kozak consensus sequence capable of translational initiation. The canonical Kozak consensus sequence is displayed with the accompanying consensus sequences flanking the human and murine sequences encoding M^1^, M^77^, and M^96^. III. Experimental confirmation of hypoxia-mediated activation of the alternate TSS in the first intron of the MMP-2 gene. Cardiomyoblast H9C2 cells were maintained in 95% or 1% O_2_ for 14 hours, followed by Western blot analysis of mitochondria-enriched fractions. The 65 kDa MMP-2 isoform is detected in fractions isolated from H9C2 cells subjected to hypoxia.

Tsuchihara, et al. [17] and Yamashita, et al. [18] have reported on the massive genome-wide physical mapping of transcriptional start sites, including 330 million tags generated by sequencing the 5′-ends of capped cDNA’s from 31 cell types and 100 million tags from cells cultured under normoxic and hypoxic conditions (DataBase of Transcription Start Sites, DBTSS). Examination of the DBTSS identified a cluster of mapped transcriptional start sites (TSS) located in the 3′ end of the first intron of the MMP-2 gene, including TSS induced by culture under hypoxic conditions (Figure 2, panel I.)

As depicted in Figure 2, panel I., transcription from the first intron skips the first MMP-2 exon which encodes the M^1^ amino acid of the full length 68 kDa MMP-2 protein. Transcription initiating within the 3′-end of the first MMP-2 intron generates a mRNA transcript in which the first methionine is located at M^77^. Cobalt (NCBI) multiple sequence alignment of the human MMP-2 coding sequence and 21 MMP-2 homologues (ranging from *Pan troglodytes* to *Xenopus laevis*) indicated the absolute conservation of three in-frame AUG’s encoding methionines at M^1^, M^77^, and M^96^ within the first 100 residues of the MMP-2 protein. Comparison of the RNA sequences flanking each AUG to the Kozak consensus sequence gccRccAUGG indicated that M^77^ and M^96^ conform to acceptable sequences for ribosomal initiation of protein synthesis and are conserved in all sequenced homologues of MMP-2 (Figure 2, panel II. aligns the human and murine MMP-2 Kozak consensus sequences).

Sequence analysis of all deposited MMP-2 transcripts in the NCBI database identified several mRNA transcripts encoding the 65 kDa MMP-2 isoform, which directly, and independently, confirms the existence of the 65 kDa MMP-2 isoform as a discrete transcriptional product. Sequence BAE87867 from a cDNA library made from macaque brain and testes encodes the 65 kDa MMP-2 isoform. Sequence BAG 63035 from a cDNA library of human mesenchymal stem cells treated with trichostatin and 5-azacytidine also encodes the 65 kDa isoform, consistent with epigenetic activation of a latent MMP-2 alternative promoter. Based on the length of the 5′UTR’s of these transcripts it is possible to precisely localize the transcriptional start sites of these two transcripts to the 3′ region of the first intron of the MMP-2 gene (Figure 2, panel I.), confirming the observations obtained with the DBTSS analysis detailed above.

We directly confirmed the activity of the hypoxia-induced TSS in the first MMP-2 intron reported in the DBTSS. Cardiomyoblast H9C2 cells were cultured overnight in either 95% or 1% O_2_, followed by gelatin affinity capture of mitochondrial lysates and Western blot analysis for MMP-2. As depicted in Figure 2, panel III., culture under hypoxic conditions generated the 65 kDa MMP-2 isoform, as predicted.

**Figure 3:**
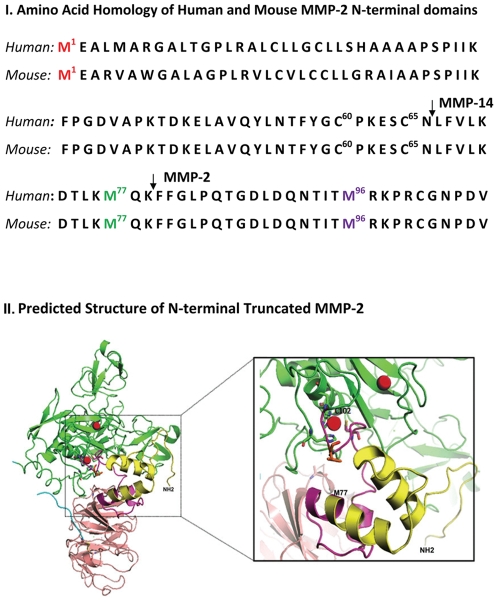
N-terminal domain MMP-2 homologies and structural analysis. I. Amino acid homology of human and mouse MMP-2 N-terminal domains. There is a high degree of amino acid homology within this domain, including conservation of methionine residues at 77 and 96 relative to the first translational start site. The methionine at position 5 in the human sequence is not conserved. M^1^, M^77^, and M^96^ are conserved in all genomic MMP-2 sequences extending to *Xenopus leavis.* Arrows denote MMP-14 (MT1-MMP) activating cleavage site at N^66^/L^67^ and MMP-2 autocatalytic cleavage site at K^79^/F^78^. II. Predicted structure of N-terminal truncated MMP-2: Overall and detailed view of human MMP-2 structure (NCBI PDB code 1CK7). Yellow: N-terminal region deleted in the NTT-MMP-2 protein; magenta: remnant of propeptide present in the NTT-MMP-2 protein; green: catalytic domain; blue: hinge region; pink: hemopexin domain; red spheres: zinc atoms. Deletion of the MMP-2 prodomain exposes the catalytically active zinc and generates the active enzyme.

Initiation of MMP-2 translation at M^77^ generates an N-terminal truncated (NTT) isoform with a predicted molecular mass of 65 kDa; initiation of translation from M^96^ generates an N-terminal truncated isoform with a predicted molecular mass of 62.5 kDa. These conform to the relative molecular masses of the mitochondria-associated MMP-2 depicted in Figure 1. The N-terminal truncated MMP-2 isoform lacks the signal sequence directing extracellular secretion. Analysis of the crystal structure of MMP-2 indicates that loss of the first 76 amino acids eliminates two of the three alpha helices that constitute the prodomain which maintains the latency of pro-MMP-2 (Figure 3, I., ref. 4). Specifically, a disulfide bridge between Cys^60^ and Cys^65^ is absent, as are prodomain-stabilizing internal hydrogen bonds [4], [19]. The M^77^residue is immediately downstream of the Asn^66^-Leu^67^ MT1-MMP cleavage site which generates active MMP-2 by destabilizing prodomain structure (Figure 3, I) [20]. Thus, the N-terminal truncated MMP-2 protein is intracellular and has intrinsic proteolytic activity due to disruption of the prodomain structure. We confirmed the proteolytic activity of recombinant N-terminal truncated MMP-2 by gelatin zymography (data not shown).

### *In Vitro* Analysis of 65 kDa MMP-2 Isoform Cellular Trafficking

**Figure 4:**
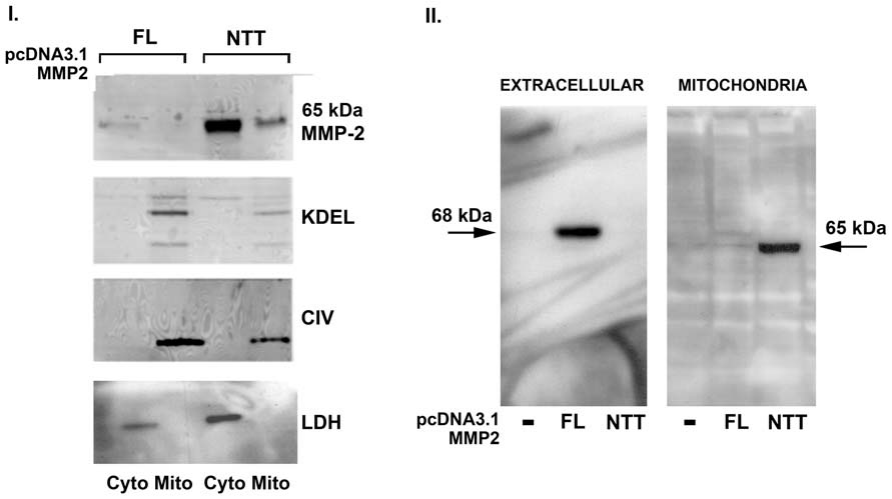
Selective cellular trafficking of 68 **kDa and 65** **kDa MMP-2 isoforms in model H9C2 cells.** I. Relative distributions of 68 (FL) and 65 kDa MMP-2 (NTT) isoforms in cytosolic and mitochondrial fractions following transient transfection with the respective expression plasmids. The quality of the cytosolic and mitochondrial fractions was assessed by Western blots for KDEL (endoplasmic reticulum), CIV (Complex IV, mitochondrial matrix) and LDH (lactate dehydrogenase, cytosol). A faint band of the 68 kDa MMP-2 isoform is present in the cytosolic fraction of cells transfected with 68 kDa MMP2 cDNA, but not in the mitochondria-enriched fraction. The 65 kDa MMP-2 isoform is present in both the cytosolic and mitochondrial fractions of cells transfected with the NTT-MMP-2 cDNA with a ratio of approximately 3∶1. The mitochondrial fractions did include faintly detectable KDEL bands, consistent with the presence of the mitochondria-associated endoplasmic reticulum in the preparation. II. H9C2 cells were transiently transfected with an empty pcDNA3.1 expression plasmid (-) or expression plasmids encoding either the 68 kDa (**F**ull **L**ength) or **N**-**T**erminal **T**runcated 65 kDa MMP-2. Western blot of the extracellular (conditioned medium) fraction revealed the 68 kDa FL, secreted isoform of MMP-2, while the NTT isoform was not detected. The FL 68 kDa isoform was not detected in mitochondria-enriched fractions, while the 65 kDa NTT isoform was present in this fraction.

To directly test the hypothesis that a truncated transcript lacking the initiator methionine would encode a 65 kDa MMP-2 protein, we used a pcDNA3.1 plasmid expressing a MMP-2 cDNA construct starting at base pair +81 relative to the ATG encoding M^1^ of the full length MMP-2 protein. The native Kozak consensus sequence flanking amino acid M^77^ (aagAagA_+229_TGc) was not modified. Transient transfections of cardiomyoblast H9C2 cells with plasmids encoding either the full length 68 kDa MMP-2 or the truncated 65 kDa MMP-2 were performed. Cytosolic and mitochondria-enriched fractions were prepared and analyzed by Western blot for MMP-2 and for markers of cyotosol (LDH), endoplasmic reticulum (KDEL) and mitochondria (Complex IV). Representative results are shown in Figure 4, panel I. Minimally detectable amounts of full length 68 kDa MMP-2 protein were present within the cyosolic fractions of H9C2 cells transfected with an expression plasmid encoding the full-length 68 kDa MMP-2 protein and none was detected within the mitochondria-enriched fraction. Cells transfected with the NTT-MMP-2 cDNA expression plasmid contained a 65 kDa MMP-2 protein present in both the cytosolic and mitochondria-enriched fractions in a ratio of approximately 3-4∶1.

To exclude the possibility that the intracellular 65 kDa MMP-2 isoform is derived from aberrant processing, proteolytic or otherwise, of the full-length 68 kDa MMP-2, we transfected H9C2 cells with the cDNA’s encoding either the full-length 68 kDa MMP-2 or the 65 kDa NTT-MMP-2 isoforms and examined the extracellular (conditioned medium) and mitochondria-enriched compartments (Figure 4, panel II). Full-length 68 kDa MMP-2 protein was readily detected in the extracellular (secretory) compartment, but not within the mitochondria-enriched fractions. In contrast, the 65 kDa NTT-MMP-2 protein was not present in the extracellular compartment but was present within the mitochondria-enriched fractions.

These experiments demonstrate that the native, non-modified Kozak consensus sequence flanking M^77^ is sufficient to serve as a translation initiation site and that the sequence +81 to +229 bp is a functional 5′ UTR. In some experiments we detected small amounts of a MMP-2 band with an apparent molecular mass of 62 kDa, suggesting that the Kozak consensus sequence flanking M^96^ is only weakly utilized in the presence of an intact M^77^ Kozak sequence. Equally important, these experiments demonstrate the selective cellular trafficking of the full-length and N-terminal truncated MMP-2 isoforms and indicate that there is no significant overlap in the cellular fates of these proteins. These studies also indicate that approximately one-third of the 65 kDa MMP-2 protein derived from transfection of the NTT-MMP2 cDNA into cardiomyoblast H9C2 cells is associated with mitochondria. Analyses using graded digitonin extraction of isolated mitochondria are consistent with localization of the 65 kDa MMP-2 protein within the mitochondrial intermembranous space (data not shown).

### The 65 kDa N-Terminal Truncated MMP-2 Protein Activates Acute Stress Signaling Cascades

Zaidi, et al. [21], first described “retrograde” calcium signaling, i.e. mitochondrial-to-nuclear signaling, initiated in response to mitochondrial DNA damage. Subsequent studies demonstrated that uncoupling of oxidative phosphorylation led to the release of mitochondrial calcium with activation of calcineurin/NFAT (Nuclear Factor of Activated T cells) and NF-κB signaling cascades [22], [23]. We postulated that MMP-2-mediated proteolysis of discrete mitochondrial proteins could trigger activation of the transcription factors, NFAT, NF-κB or interferon response factors (IRF1/7), and thereby induce nuclear gene transcription.

**Figure 5:**
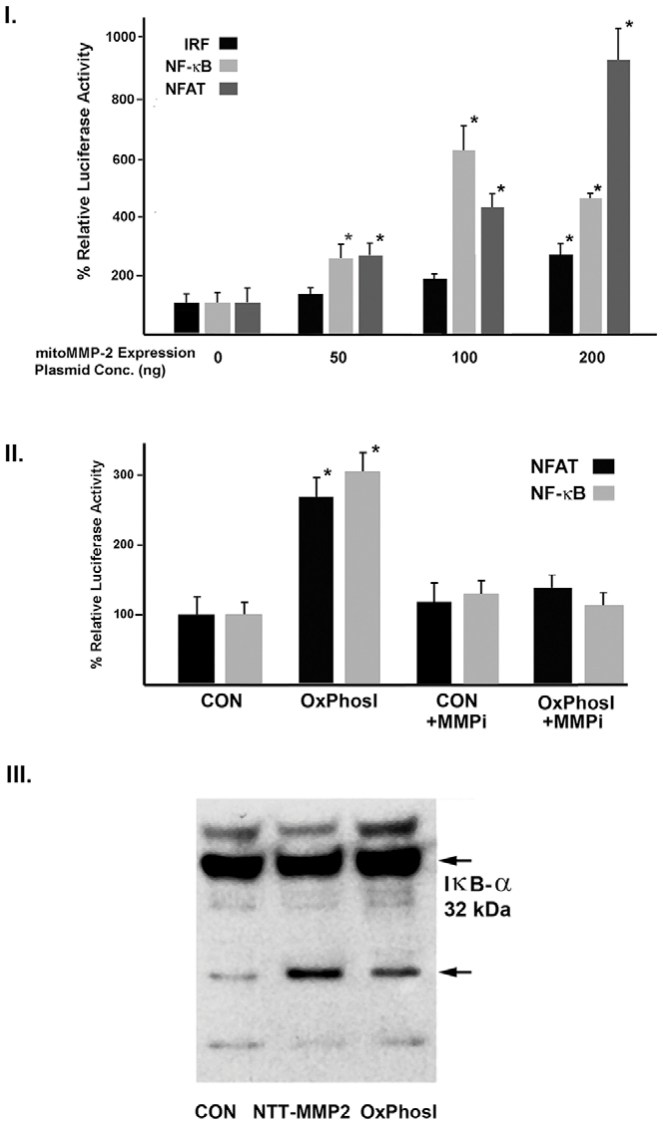
The N-terminal truncated MMP-2 and activation of stress-signaling cascades. I. H9C2 cells were transfected with increasing concentrations of the NTT-MMP-2 expression plasmid, along with luciferase reporter plasmids for NFAT, NF-κB and IRF (interferon response factor). NTT-MMP-2 enhances inflammatory transcriptional signaling in a concentration-dependent manner (*P<0.05). II. Transient OxPhosI induces activation of NFAT and NF-κB signaling: dependence on MMP-2 activity. H9C2 cells were transfected with NFAT and NF-κB luciferase reporter plasmids and subjected to transient OxPhosI as detailed in Materials and Methods, in the presence or absence of the cyclic peptide MMP-2 inhibitor, CTTHWGFTLCGG (25 µM). III. NTT-MMP-2 degrades mitochondria-associated IκB-α. H9C2 cells were subjected to either transient OxPhosI or transfected with the NTT-MMP-2 expression plasmid. Thereafter the mitochondria were isolated, solubilized and Western blots performed for NF-κB inhibitory IκB-α. Degradation peptide fragments denoted with arrow.

We transiently transfected H9C2 cells with luciferase reporter constructs containing concatenated response elements for interferon response factors (IRFs), NF-κB or NFAT. The cells were cotransfected with a control expression plasmid or with increasing concentrations of the N-terminal truncated MMP-2 cDNA. As shown in Figure 5, panel I., transfection of the N-terminal truncated MMP-2 cDNA resulted in significant increases in luciferase reporter activity for all three transcription factors in a concentration-dependent manner. The relative increases were ranked NFAT>NFκB>>IRF. These experiments indicate that the 65 kDa N-terminal truncated MMP-2 can activate stress signaling, particularly by NFAT and NFκB.

To determine if the activation of mitochondrial-to-nuclear signaling by N-terminal truncated MMP-2 were dependent on MMP-2 proteolytic activity, H9C2 cells were subjected to OxPhosI in the presence or absence of 50 µM of the selective MMP-2 cyclic peptide inhibitor, CTTHWGFTLCGG [24]. As anticipated, OxPhosI stimulated both NFAT and NFκB signaling.

Inclusion of the MMP-2 inhibitor blocked OxPhosI-induced NFAT and NF-κB transcriptional activation, indicating that N-terminal MMP-2 proteolytic activity is required for this process (Figure 5, panel II.).

### The 65 kDa N-Terminal Truncated MMP-2 Mediates Proteolysis of Inhibitory IκB-α

NF-κB is complexed with members of the IκB family to maintain NF-κB in an inactive state [25], [26]. Cytokine activation triggers phosphorylation of IκB proteins, resulting in ubiquitination and proteasomal degradation. This allows free NF-κB to translocate to the nucleus and initiate transcription. Cogswell, et al. [27] localized NF-κB and IκB-α within mitochondria in association with the inner mitochondrial membrane. NF-κB was subsequently shown to directly interact with the adenine nucleotide translocator (ANT) and regulate mitochondrial gene expression and apoptosis [28]. Non-proteasomal degradation of IκB-α, with activation of NF-κB, has been described as well [29], [30]. To determine if the N-terminal truncated MMP-2 degrades IκB-α, we isolated mitochondria from H9C2 cells subjected to OxPhosI or following transfection with the N-terminal truncated MMP-2 cDNA, followed by Western blot analysis for IκB-α. The results of these studies are shown in Figure 5, panel III. Both OxPhosI and transfection with the N-terminal truncated MMP-2 cDNA resulted in appearance of IκB-α degradation products. In separate experiments we confirmed that recombinant MMP-2 degrades IκB-α present in isolated mitochondria (data not shown).

### The 65 kDa N-Terminal Truncated MMP-2 Induces an Innate Immune Response Transcriptome

As detailed above, the 65 kDa N-terminal truncated MMP-2 activates NFAT, NF-κB and IRF transcriptional factors, key components of the innate immunity transcriptional network. To determine the specific gene ontologies regulated by these transcription factors we performed a genome-wide transcriptional analysis of H9C2 cells transfected with the 65 kDa N-terminal truncated MMP-2 cDNA and compared these to the gene ontologies regulated by transfection with the full length 68 kDa MMP-2 cDNA. Controls were transfected with an empty pcDNA3.1 expression plasmid as detailed in Methods. The results of these studies are summarized in Tables S1, S2, and S3. Using stringent conditions of analysis 36 annotated transcripts (from a target total of 10 K) were up-regulated by 65 kDa N-terminal truncated MMP-2, while 28 annotated transcripts were down-regulated. Twenty-seven of the N-terminal truncated MMP-2 up-regulated transcripts could be assigned to ontologies with 2 ≥ components, while only twelve N-terminal truncated MMP-2 down-regulated transcripts could be assigned to ontologies with 2 ≥ components. Only six annotated transcripts were significantly up-regulated by transfection of H9C2 cells with the full-length MMP-2 construct. There were no clear ontologies in this group and no overlap with the transcripts up-regulated by N-terminal truncated MMP-2.

Five discrete ontologies could be assigned to the N-terminal truncated MMP-2 up-regulated transcripts (Table S1). The largest ontology consisted of viral stress induced genes and included ten components, including OAS1A, OAS1B, OASL1, IFIT1-3, and PRKRA. These genes are central components of a primary innate immune response and initiate cellular defense mechanisms, and also promote apoptosis [31].

The N-terminal truncated MMP-2 cDNA also induced the innate immune response transcription factors IRF7, STAT2, IFP35 and the transcriptional cofactor PARP14. Four critical innate immunity chemokines/cytokines were induced by N-terminal truncated MMP-2, including CXCL1 and CXCL10, CLL2 (MCP-1) and IL6.

Transcripts down-regulated by N-terminal truncated MMP-2 included five genes associated with resistance to apoptosis or oxidative stress, including BCLX (Bcl-xL) and HSPD1 (heart shock protein-1, chaperonin, Table S2). Heat shock protein-1 is a mitochondrial chaperone that induces resistance to stress-induced apoptosis, while Bcl-xL is one of the most important anti-apoptotic factors induced in response to cellular stress [32], [33]. Also noted is the down-regulation of five transcripts, encoding components of the contractile apparatus, including troponin I and myosin light chain-2.

Thus, NTT-MMP-2 induces transcription of a discrete gene ontology set comprised of primary innate immune response genes, pro-inflammatory cytokines and chemokines. Pro-apoptotic ontologies were upregulated, while anti-apoptotic ontologies were down regulated. The results of the microarray data for transcripts up-regulated by transfection with the N-terminal truncated MMP2 cDNA were validated by PCR analysis (Figure S3).

In stark contrast to the results obtained with the N-terminal truncated MMP-2, transfection of H9C2 cells with the full-length MMP-2 cDNA resulted in the significant induction of only six annotated transcripts and no discrete ontologies with two or more components (Table S3A). Six annotated transcripts were significantly down-regulated by transfection with the full-length MMP-2 cDNA and no discrete ontologies were identified (Table S3B). The physiologic significance of the full-length MMP-2 regulated transcripts is unclear.

### *In Silico* Analysis of Promoters of N-Terminal Truncated MMP-2 Regulated Transcripts

**Figure 6:**
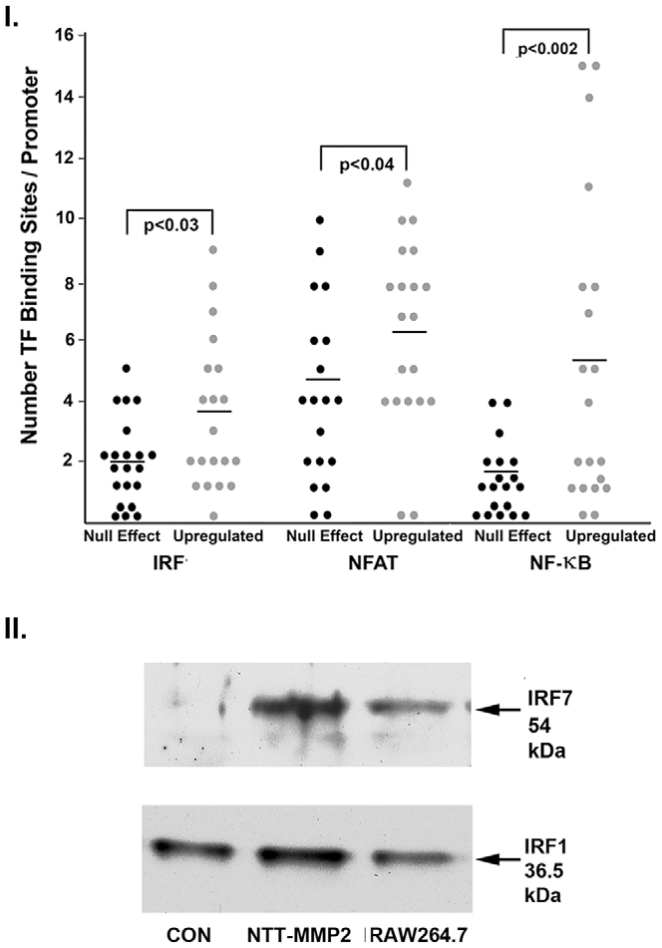
*In silico* analysis of promoters of genes upregulated by NTT-MMP-2. The frequency of cognate DNA binding motifs for IRF, NFAT and NF-κB present in 2 kb of the 20 transcripts most up-regulated by NTT-MMP-2 and of 20 randomly chosen transcripts not modified by NTT-MMP-2 were determined by database analysis. Horizontal bars depict the mean of each data set. II. Western blot for IRF7 and IRF1 of nuclear extracts prepared from H9C2 cardiomyoblast cells transfected with a control pcDNA3.1 plasmid (CON), cells transfected with the pcDNA3.1 NTT-MMP2 expression plasmid (NTT-MMP2) and an IRF7 and IRF1 positive control nuclear extract prepared from LPS-stimulated macrophage RAW264.7 cells. Transfection of NTT-MMP2 cDNA induces IRF7, but not IRF1, nuclear localization.

We examined 2 kb of the proximal promoters of the 20 genes most up-regulated by transfection of H9C2 cells with the N-terminal truncated MMP-2 cDNA for the frequency of cognate binding sites for the transcription factors IRF1/7, NFAT and NF-κB and compared these data with the frequency of identical binding sites in the proximal promoters of 20 randomly selected, non-responsive genes. The results of this analysis are summarized in Figure 6, I. The proximal promoters of N-terminal truncated MMP-2 up-regulated genes contained 3.45±2.54 IRF binding sites as compared to 1.95±1.47 binding sites in the nonresponsive promoters (P<0.03, n = 20 for each group). There were 6.25±3.13 NFAT binding sites in the responsive promoters, while 4.16±3.0 NFAT sites were present in the nonresponsive promoters (P<0.04, n = 20 for each group). NF-κB binding sites were significantly more frequent in the responsive promoters as compared to the nonresponsive promoters (5.15±5.1 vs. 1.21±1.32, P<0.002, n = 20 for each group). The total number of transcription factor binding sites (sum of IRF 1/7, NFAT and NF-κB binding sites) was 7.55±3.7 in the nonresponsive promoters and 14.8±6.9 in the responsive promoters (P<0.01). NFAT and NF-κB binding sites were generally present on the same set of responsive promoters, while the IRF 1/7 binding sites in the responsive promoters were primarily present on a second, discrete set of genes.

### NTT-MMP Promotes IRF7 Nuclear Localization

The transcriptional activity of IRF7 is affected both by absolute protein levels but more importantly by phosphorylation and nuclear translocation [34]. Nuclear extracts from control H9C2 cells transfected with the empty pcDNA3.1 expression vector and nuclear extracts from cells transfected with the N-terminal truncated MMP-2 cDNA were prepared. We probed these extracts for IRF7 by Western blot analysis. Nuclear extracts from the macrophage cell line RAW 264.7 stimulated with endotoxin were used as positive controls. As shown in Figure 6, II., IRF7 protein was below the levels of detection in the control H9C2 nuclear extracts, but was readily detected in the nuclear extracts of H9C2 cells transfected with the N-terminal truncated MMP-2 cDNA. There was no change in the nuclear concentrations of the control IRF1 protein.

## Discussion

### The 65 kDa MMP-2 Isoform is a Discrete Functional Entity

The principal finding of this study is the identification of a novel intracellular N-terminal truncated 65 kDa isoform of MMP-2 that activates an innate immune response. The 65 kDa MMP-2 isoform is structurally and functionally distinct from the sarcomeric latent full length 68 kDa MMP-2 isoform described by Schulz and colleagues [7]–[9]. The N-terminal truncated MMP-2 isoform is not present under basal conditions and is generated under conditions of oxidative stress both *in vitro* and *in vivo*. The cellular trafficking of the N-terminal truncated isoform is clearly distinct from the full length MMP-2. The N-terminal truncated isoform is present in both the cytosol and mitochondrial fractions. The MMP-2 protein does not include a canonical mitochondrial targeting sequence and the precise mechanism whereby a proportion of the 65 kDa isoform enters mitochondria remains to be explored. As recently reviewed, there are additional mechanisms, including chaperone proteins, that regulate mitochondrial protein import that do not require a N-terminal targeting sequence [35]. Heat shock protein 90 is an important intracellular chaperone associated with transport of client proteins to the mitochondria and has been recently shown to physically interact with MMP-2 [36], [37]. This awaits direct experimental verification, but suggests that MMP-2 may enter mitochondria as a client protein of specific cellular chaperones.

### Mechanisms of 65 kDa MMP-2 Isoform Generation

We considered several potential mechanisms for the generation of the 65 kDa isoform. Firstly, physiologic processing of the 68 kDa proenzyme takes place in the extracellular space and has been fully characterized [4], [19], [20]. Pro-MMP-2 (68 kDa) is secreted and forms a complex on the cell surface with MT1-MMP and TIMP2. MT1-MMP cleaves the Asn^66^-Leu^67^ bond in the MMP-2 prodomain, followed by autocatalytic cleavage to generate the 62 kDa active extracellular MMP-2 protein. The transfection studies detailed in Figure 4 clearly demonstrate the separate cellular targeting pathways of full-length 68 kDa MMP-2 vs. the 65 kDa isoforms. Finally, it was not possible to generate a 65 kDa MMP-2 protein by transfection with the cDNA encoding the 68 kDa isoform.

In terms of alternative splicing, the European Bioinformatics Institute Alternative Splicing and Transcript Diversity (ASTD) database (www.ebi.ac.uk/astd) assembled a total of seven human MMP-2 transcripts (including the full-length reference transcript). One transcript has a slightly shorter 5′UTR and would be predicted to encode the full-length MMP-2 protein. Five transcripts are extensively truncated at the C-terminus, presumably due to premature termination of transcription, and all encode putative proteins of less than 50 kDa in size.

A third mechanism for the generation of protein diversity results from the activation of alternative promoters. This is frequently coupled with the use of alternative translational start sites, thereby generating N-terminal truncated protein variants. A recent bioinformatic analysis of the human transcriptome indicates that N-terminal in-frame methionines associated with functional Kozak consensus sequences are not rare and employment of this mechanism is responsible for the N-terminal modification of several key regulatory proteins, including p53, c-myc, osteopontin and renin, among others [38]–[41].

### Role of the First Intron in the Expression of the 65 kDa MMP-2 Isoform

We have previously reported that the first intron of the MMP-2 gene plays a critical role as a regulator of MMP-2 transcription following in vivo ischemic injury [42]. We demonstrated that the first intron has an enhancer function and was required for increased MMP-2 transcription mediated by NFATc2 binding to the 3′ region of the intron. Analysis of physically mapped MMP-2 transcriptional start sites and deposited MMP-2 transcripts encoding the 65 kDa MMP-2 isoform identify the first intron of the MMP-2 gene as an alternative promoter, as well. Based on the ENCODE project epigenetic mark and chromatin structure analyses, [43], the first intron of the MMP-2 gene includes DNAse hypersensitivity sites, chromatin structures consistent with a poised promoter and the histone marks, H3K4Me3 and H3K4Me1, characteristic of active promoters and enhancers, respectively (Figure 2, panel I.).

A recent genome-wide study of the effects of hypoxia on activation of alternative promoters identified a hypoxia-inducible alternative MMP-2 promoter localized to the 3′ region of the first intron [17]. We directly confirmed this observation within the context of hypoxic H9C2 cells. Hypoxia, or accompanying redox stress, can promote genomic DNA demethylation, thereby permitting activation of intragenic (intronic) alternative promoters [44], [45]. Yamashita, et al. [18] provided a quantitative assessment of the relative transcript abundance of the full length and 65 kDa MMP-2 isoforms in a variety of adult and fetal tissues and cell lines. The transcript encoding the 65 kDa MMP-2 isoform was not detected in templates derived from adult tissues, but was detected in fetal heart templates and templates from cultured fetal lung fibroblasts cultured under hypoxic conditions. In the current study, we employed the H9C2 cardiomyoblast cell line which is derived from embryonic rat heart [46]. Taken together, these observations suggest that expression of the 65 kDa MMP-2 isoform may be a component of the fetal gene re-expression program characteristic of cardiac hypertrophy and failure [47].

### Activation of Nf-κB and NFAT Signaling and Expression of a Pro-Inflammatory, Pro-Apoptotic Transcriptome

We determined that transient generation of redox stress was associated with activation of NF-κB and NFAT signaling cascades, an event dependent upon MMP-2 enzymatic activity. Direct demonstration that the 65 kDa isoform activates inflammatory signaling cascades was provided by transfection studies using the 65 kDa MMP-2 cDNA. One potential explanation for the activation of these signaling cascades is provided by the studies demonstrating that mitochondrial IκB-α is degraded by MMP-2, thereby freeing NF-κB for transport to the nucleus. Given the finding that the 65 kDa MMP-2 isoform was also present in the cytoplasm, it is conceivable that NF-κB activation takes place in this compartment as well.

Microarray analysis revealed that the 65 kDa MMP-2 isoform induced a remarkably focused set of innate immunity gene ontologies, the promoters of which were highly enriched in binding sites for NFAT and NF-κB. These gene ontologies have been primarily reported in association with viral infection and generally promote apoptosis and recruitment of inflammatory cells. The 2′–5′-oligoadenylate synthetases (OAS1A/B, OASL1) synthesize 2′,5′-linked phosphodiester bonds to polymerize ATP into adenosine oligomers and thereby activate latent RNAse L [31], [48]. Active RNAse L degrades both viral and cellular RNA’s, including intact cellular 28S rRNA, resulting in inhibition of viral and cellular protein synthesis, with consequent growth inhibition and cellular apoptosis.

The interferon-induced proteins with tetratricopeptide repeats 1–3 (IFIT1–3) interfere with ribosomal assembly and block interaction with eIF3 subunits, thereby inhibiting translation of viral and cellular proteins [49]. IFIT proteins also inhibit protein translation via interaction with eukaryotic elongation factor-1A [49]. Protein kinase, interferon-inducible double stranded RNA-dependent (PRKRA) is a serine/threonine kinase that phosphorylates the eIF2α translation initiation factor in response to stress signals, thereby arresting viral and cellular protein synthesis [50]. PRKRA activation subsequently leads to cellular growth inhibition and apoptosis. Adenosine deaminase RNA-specific (ADAR) is an RNA-specific editing enzyme that converts adenosine to inosine with RNA and is involved in editing of specific pre-mRNA transcripts affecting a number of cellular processes, including the balance between pro- and anti-apoptotic factors [51], [52].

The 65 kDa MMP-2 upregulated IRF7, which is the master regulator of type-I interferon-dependent immune responses and exerts transcriptional control over a large set of pro-inflammatory genes [34]. STAT2 is critically involved in the signal transduction of several pro-inflammatory signaling cascades.

Upregulated chemokines and cytokines included chemokine (C-X-C motif) ligand-1 (CXCL1) and chemokine (C-X-C motif) ligand-10 (CXCL10), which are chemotactic for neutrophils and monocyte/T cells, respectively. Chemokine (C motif) ligand-2 (MCP-2, monocyte chemotactic protein-1) is one of the most studied factors leading to cardiovascular disease and plays a major role in myocarditis, ischemia/reperfusion injury and cardiomyocyte death [53]–[55]. IL6 plays a significant role in the induction of cardiomyocyte hypertrophy and inflammatory signaling and is a predictor of outcome in clinical heart failure [56], [57].

The *in vitro* experiments support a model whereby N-terminal truncated MMP-2 generated by redox stress or hypoxia triggers pro-inflammatory signaling cascades by NF-κB, NFAT and to a lesser extent IRF. This stress signaling lead to the transactivation of a discrete set of genes linked to the innate immune response.

### Relationship of N-Terminal Truncated MMP-2 to Other MMP-2 Isoforms

MMP-2 plays multifaceted roles in cardiac disease and the current study adds yet another, and unexpected, level of complexity. The experimental and clinical evidence supporting a major role for MMP-2 in cardiac disease, is well documented [1], [10], [11], [58]–[63]. In these investigations the emphasis has been on the presumed extracellular actions of MMP-2. The present study, in conjunction with the reports of Schulz and colleagues, [6]–[9], strongly support the concept that the intracellular activities of discrete MMP-2 isoforms may also be of great pathophysiologic importance.

**Figure 7:**
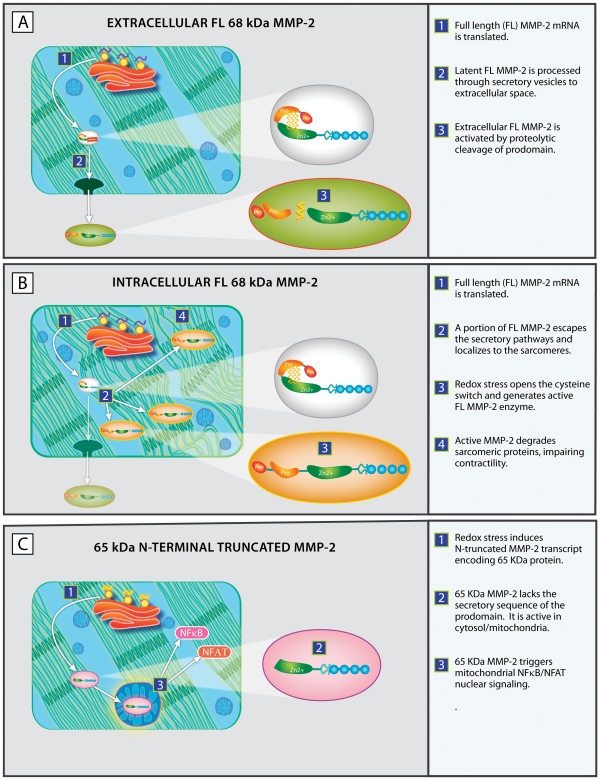
Schematic detailing the distinctive processing, localization and activation mechanisms of three known isoforms of MMP-2. Upper Panel: The mRNA transcript for the full length 68 kDa MMP-2 protein is translated and the latent, enzymatically inactive MMP-2 protein is processed through the Golgi and secretory vesicles to the extracellular space. Latent MMP-2 protein is activated by proteolytic cleavage, primarily by MT1-MMP. This removes the inhibitory prodomain, yielding active 62 kDa MMP-2 protein in the extracellular space where the enzyme degrades extracellular matrix components. Middle Panel: The mRNA for the full length 68 kDa protein is translated and a fraction of the synthesized latent MMP-2 protein escapes the secretory pathway and localizes specifically to sarcomeric proteins, including troponin I. Transient redox stress, such as is induced by ischemia reperfusion injury, generates reactive oxygen species and peroxynitrites which open the cysteine switch. This produces active full length MMP-2 which degrades several components of the sarcomeric apparatus, leading to impaired contractility. Lower Panel: Hypoxia and redox stress activate a latent promoter in the first intron of the MMP-2 gene, thereby generating a N-terminal truncated mRNA transcript. The translated 65 kDa MMP-2 isoform lacks the secretory sequence and inhibitory prodomain and is enzymatically active in the cyotosol and mitochondria. The 65 kDa MMP-2 isoform activates NF-κB and NFAT mitochondrial-nuclear stress signaling, which induction of a pro-inflammatory, pro-apoptotic transcriptome.

Figure 7 is a schematic which places the various MMP-2 isoforms expressed by cardiomyocytes into their respective cellular contexts. As depicted in the upper panel, “classical” extracellular MMP-2 is translated from a full length mRNA transcript and is exported to the extracellular space via secretory vesicles in the latent, or inactive, form. MMP-2 activation occurs through the proteolytic removal of the inhibitory prodomain, most commonly performed by MT1-MMP complexed with TIMP2. Active MMP-2 thereafter participates in the turnover of extracellular matrix components and basal laminae.

As shown in the middle panel, intracellular full length latent MMP-2 is translated from a full length mRNA transcript. As recently reported [64], a fraction of the newly synthesized protein escapes from the secretory apparatus and localizes to cardiomyocyte sarcomeres, where it remains in a latent, or inactive, form. Ischemia/reperfusion injury generates reactive oxygen species and peroxynitrite which open the cysteine switch. This generates active MMP-2 in which the inhibitory prodomain no longer covers the zinc-containing catalytic site. Active MMP-2 then degrades sarcomeric proteins, resulting in impaired contractility.

The lower panel summarizes the distinctive features of the 65 kDa N-terminal truncated MMP-2 isoform which clearly distinguish it from the MMP-2 forms discussed in the upper and middle panels. The 65 kDa MMP-2 isoform is not found under basal conditions. Redox stress induced by hypoxia or ischemia/reperfusion injury activates a latent promoter in the first intron of the MMP-2 gene, generating a truncated mRNA transcript encoding the N-terminal truncated 65 kDa MMP-2 isoform. This isoform lacks the secretory signal sequence and inhibitory prodomain and is present in the cytosol and mitochondrial as an active enzyme. The 65 kDa MMP-2 isoform degrades inhibitory IκB-α, thereby activating NFκB/NFAT mitochondrial-nuclear stress signaling with induction of an innate immunity transcriptome.

### Summary and future directions

In this report we have defined a previously unrecognized intracellular isoform of MMP-2 that is induced by hypoxia and oxidative stress by activation of a latent promoter in the first intron of the gene. We have recently extended the primarily in vitro observations detailed in this study by completing an analysis of transgenic mice with cardiac-specific expression of the N-terminal truncated 65 kDa MMP-2 isoform (Lovett, et al., unpublished observations). As predicted by the current studies, the mice develop significant cardiomyocyte and ventricular hypertrophy associated with progressive systolic failure, cardiomyocyte apoptosis and inflammatory cell infiltration. Further, the mice exhibit increased injury following ischemia/reperfusion injury. Recent degradomic analyses indicate that MMP-2, in contrast to MMP-9, has an extensive (>200) number of discrete substrates, many of which are intracellular [65], [66]. Ongoing proteomic studies of the N-terminal truncated MMP-2 transgenic mice may be expected to provide new mechanistic insights into the pathogenesis of cardiac disease, particularly in the setting of acute or chronic oxidant stress.

## Materials and Methods

### Cardiac MMP-2 Western Blot Analyses

All murine studies were approved (protocol 09-053-03) by the Animal Care Committee of the San Francisco VAMC Animal Care and Use Committee (IACUC). This institution is accredited by the American Association for the Accreditation of Laboratory Animal Care (Assurance Number A3476-01). Freshly isolated hearts were homogenized in 0.25 M sucrose, 10 mM HEPES, pH, 7.5, 5 mM EDTA and protease inhibitor cocktail at 4°C, followed by centrifugation at 700 g for 5 minutes. The supernate was centrifuged for an additional 5 minutes at 700 g and the supernate centrifuged at 9000 g for 5 minutes to pellet mitochondria. The mitochondrial pellets were washed twice in isolation buffer, followed by a final wash in isolation buffer containing 75 mM KCl. Mitochondrial-enriched pellets were homogenized in lysis buffer (50 mM Tris/HCl, pH 7.4, 150 mM NaCl, 0.5% Triton X-100, 0.5% CHAPS, 0.5% sodium deoxycholate, plus protease inhibitor cocktail), sonicated briefly on ice and the supernate collected after centrifugation at 10,000 g for 20 minutes. The cleared mitochondrial extracts (150 µg protein/sample) were incubated overnight at 4°C with 100 µl gelatin-Sepharose beads (Sigma-Aldrich) in 500 µl 50 mM Tris/HCl, pH 7.4 to affinity absorb MMP-2. Thereafter, the beads were washed three times in binding buffer, followed by elution in an equal volume of 2 X SDS-PAGE sample buffer. Protein electrophoresis was performed with the NuPAGE Bis-Tris gel system with MOPS buffer (Invitrogen). Western blots used murine monoclonal anti-MMP-2 (Ab-3, Calbiochem) followed by HRP-conjugated goat anti-mouse IgG (Zymed) and detection with ECL-Plus reagent.

### Cell Culture, Induction of Graded Inhibition of Oxidative Phosphorylation (OxPhosI), Hypoxia Treatment

Cardiomyoblast H9C2 cells were obtained from ATCC (Rockville, MD) and maintained in DMEM supplemented with 4 mM L-glutamine, 1.6 gm/L sodium bicarbonate, 4.6/L glucose and 10% fetal bovine serum (complete DMEM). Inhibition of oxidative phosphorylation FIX was modified from the protocol of Cybulsky, et al. [15]. For partial inhibition of oxidative phosphorylation, H9C2 cells were incubated with DMEM lacking pyruvate and D-glucose for 15 minutes at 37°C. Thereafter, the cells were washed, given complete medium and cultured for an additional 24 to 72 hours, prior to cell fractionation. For more complete inhibition of oxidative phosphorylation, washed H9C2 cells were incubated in pyruvate/D-glucose-free DMEM containing 2 µM antimycin A, an inhibitor of mitochondrial Complex III, and 2-deoxyglucose (5 mM) for 15 minutes. Thereafter, the cells were washed and incubated in complete DMEM for 24 to 72 hours, followed by fractionation and Western blot analysis as detailed above.

To determine the effects of hypoxia, subconfluent cultures of H9C2 cells were cultured in complete DMEM for 14 hours at 1% O_2_ in a hypoxia chamber prior to harvesting, fractionation and Western blot analysis as detailed below.

### H9C2 Mitochondria/Cystosolic Fractionation

All steps were performed at 4°C in the presence of protease inhibitor cocktail (Pierce). Cultures were washed in calcium/magnesium-free PBS (CMF-PBS), harvested and pelleted in CMF-PBS by centrifugation at 500 g for 10 minutes. The cellular pellets were suspended in 10 mM NaCl, 1.5 mM MgCl_2_, 10 mM Tris/HCl, pH 7.5, for 10 minutes at 4°C, followed by Dounce homogenization in 210 mM mannitol, 70 mM sucrose, 5 mM Tris/HCl, pH 7.5, 1 mM EDTA (XMS solution) plus protease inhibitors (Pierce). The homogenate was centrifuged at 800×g for 20 minutes and the resulting supernate recentrifuged twice using the same conditions. This supernate was then centrifuged at 19,000×g for 15 minutes to pellet mitochondria, which were subsequently washed twice with XMS under the same conditions. The supernate from the first centrifugation step was centrifuged at 100,000×g for 15 minutes to obtain a cytosolic fraction. The respective fractions were analyzed by Western blot for the cytosolic marker LDH, (goat-anti-LDH, Abcam, 1 µg/ml), the endoplasmic reticulum marker, KDEL (rabbit anti-KDEL, Abcam, 2 µg/ml) and the mitochondrial marker, Complex IV (monoclonal murine IgG anti-Complex IV, subunit 1, Molecular Probes, 0.5 µg/ml), followed by secondary rabbit anti-goat-IgG-HRP (Zymed), goat anti-rabbit IgG-HRP (Zymed) or goat anti-mouse IgG-HRP (Zymed), respectively. Western blots for MMP-2 were performed using the gelatin affinity capture technique detailed above.

### Construction of N-Terminal Truncated (NTT) MMP-2 cDNA

The cDNA encoding the full-length human MMP-2 protein was obtained from Origene. Using the full length MMP2 cDNA as a template, NTT-MMP2 cDNA was generated with sense primer, 5′-TGCAAGCTT-TTGTGCTGAAAGATACC3′, and antisense primer, 5′-CCTCTAGACTCGAGCGGC-3′. This generated an NTT-MMP-2 cDNA construct (cloned into pcDNA3.1, Invitrogen) starting at base pair +81 relative to the ATG encoding M^1^ of the full length MMP-2 protein. The native Kozak consensus sequence flanking amino acid M^77^ (aagAagA_+229_TGc) was not modified.

An NTT-MMP-2 protein positive control was generated by transient transfection of the NTT-MMP-2 cDNA expression plasmid into CHO cells (ATCC) using standard methodology. At 48 hours following transfection, CHO cells were lysed into 50 mM Tris/HCl, pH 7.4, 150 mM NaCl, 0.5% Triton X-100, 0.5% CHAPS, 0.5% sodium deoxycholate, plus protease inhibitor. The NTT-MMP-2 protein was recovered by affinity chromatography on gelatin-coupled Sepharose (Sigma) as reported [67]. A full-length MMP-2 positive control protein was generated in a similar fashion from CHO cell conditioned medium.

### Selective Trafficking of Full Length-MMP-2 and NTT-MMP-2

Subconfluent H9C2 cultures were transiently transfected for 48 hours in serum-free DMEM with control, empty pcDNA3.1 plasmid, the plasmid encoding full-length MMP-2 cDNA or the NTT-MMP-2 plasmid (200 ng/ 60 mm dish). Mitochondrial fractions were prepared as detailed above. The conditioned medium from the three study groups was collected and centrifuged at 10,000 g for 20 minutes. The mitochondrial and extracellular fractions were analyzed by Western blot for MMP-2 as detailed above.

### Luciferase Reporter Assays

Luciferase reporter plasmids containing concatenated enhancer binding sites for the interferon response factor (IRF, pHTS-IRF), Nuclear Factor of Activated T-cells (NFAT, pHTS-NFAT) and NK-κB (pHTS-NF-κB) were obtained from Biomyx. Subconfluent H9C2 cells were washed and transiently transfected with Fugene 6 using 200 ng/well of the pHTS-NFAT and pHTS-NF-κB reporter plasmids. At 24 hours the H9C2 cells were subjected to the full OxPhosI protocol, including antimycin and 2-deoxyglucose. Controls were maintained in complete medium. Luciferase activity (Luciferase Assay System, Promega) was measured after a further 48 hours of culture in complete medium. The requirement for MMP-2 activity for induction of luciferase activity was assessed by inclusion of the cyclic peptide MMP-2 inhibitor, CTTHWGFTLCGG (Calbiochem) at 25 µmol/l. Values are given as means±SD of triplicate to quadruplicate measurements of relative luciferase activity wherein controls are assigned a luciferase activity of 100%.

To determine if N-terminal truncated MMP-2 induced inflammatory signaling cascades in the absence of OxPhosI, H9C2 cells were transiently transfected with 200 ng of the respective pHTS reporter plasmids in the presence of increasing concentrations of the NTT-MMP-2 pcDNA expression plasmid (0-200 ng plasmid DNA/dish). Luciferase activity was determined at 48 hours and expressed as detailed above.

### Proteolytic Degradation of IκB-α

H9C2 cells were subjected to complete OxPhosI as detailed above. After 48 hours mitochondria were isolated and Western blots for Iκ-Bα performed (rabbit anti-IκB-α IgG, Santa Cruz Biotechnologies, 1∶1000, followed by goat-anti-rabbit IgG/ECL-Plus). H9C2 cells were also transiently transfected with NTT-MMP-2 cDNA (200 ng/dish) followed by mitochondrial isolation at 48 hours and Western blot analysis for Iκ-Bα.

### Microarray Analysis

Subconfluent cultures of H9C2 cells were transfected for 48 hours with a control, empty pcDNA3.1 plasmid, NTT-MMP-2 pcDNA, or full-length MMP-2 pcDNA (200 ng plasmid DNA/60 mm dish). RNA from six plates for each plasmid was extracted with TRIzol, quality analyzed (Agilent 2100 Bioanalyzer) and pooled, followed by repeat quality analysis. Target RNA was prepared with the MessageAmp cRNA amplification kit (Ambion), quality assessed and hybridized with the 10 K Uniset Rat microarray according to the manufacturer’s instructions (Codelink Gene Expression System, GE Health Care). Microarray data were normalized, transformed to the log_2_ (GenePix4000) and analyzed with the GeneSifter software package (Geospiza). Data analysis parameters included greater than 2-fold change in expression level, a quality call of 1, a P value of <0.01 and correction for multiple comparisons using the Benjamini and Hochberg algorithm.

### PCR Validation of Microarray Results

RNA was prepared (TRIzol) from H9C2 cells 48 hours after transfection with a control, empty pcDNA3.1 plasmid or after transfection with NTT-MMP-2 pcDNA using the same conditions detailed above and quality assessed with the Agilent 2100 Bioanalyzer. cDNA templates were generated by oligo-dT priming (Transcriptor, Roche, Alameda, California). Polymerase chain reactions were performed (Agilent 9800) and normalized to housekeeping GAPDH transcript levels with the following primer pairs:

GAPDH: 5′-TGACATCAAGAAGGTGGTGAAGCAGGCAT-3′/5′-CACCCTGTTGCTGTAGCCGTATTCATTGTCAT-3

OAS1: 5′-caagcactggtaccaactgtg-3′/5′-CTCCAGGGCGTACTGTGG-3′

OASL1: 5′-CAGTCATTGAGCGCTTCGT-3′/5′-CTGCTGGGTCCAGGATAATG-3′

IRF7: 5′-CCCAAGGAGAAGAGCCTGAT-3′/5′-GCCTTCCAGATGTGTCTTGC-3′

CXCL1: 5′-CACACTCCAACAGAGCACCA-3′/5′-TGACAGCGCAGCTCATTG-3′

STAT2: 5′-CACTTGAAGGATTGGAAGTTGA-3′/5′-GCGCCATTTGGACTCTTC-3′

IFIT1: 5′-CTTTGCTGAAATGCCACGTA-3′/5′-GGATCACGAGAGCCATAAAGA-3′

IFIT3: 5′-GGAAGAACTGAGAAGATTAACTATGGA-3′/5′-GGGAAATCGAT-GAGGTCTGA-3′

IRF9: 5′-AGGACCCAGTGTTCATGGAG-3′/5′-GGTGAGCAGCAGCGAGTAGT-3′

### *In Silico* Analysis of MMP-2 Regulated Promoters

Two kilobases of the proximal promoters of the twenty transcripts most up-regulated by transfection with NTT-MMP-2 were retrieved. As controls, two kilobases of twenty randomly selected, unregulated transcripts were also retrieved. The number of consensus transcription factor binding sites for IRF7, NFAT and NF-κB were determined in each promoter using the Transcription Element Search System (www.cbil.upenn.edu/tess) and the TRANSFAC v6.0 and JASPAR databases. Data are expressed as the number of transcription factor binding sites/promoter and compared using an unpaired t-test, with P<0.05 considered significant.

### Western Blot Analysis of Nuclear IRF7 and IRF1

To determine if NNT-MMP2 induces IRF7 transcriptional activation through induction of IRF7 nuclear localization, as compared to control IFR1, H9C2 cells were transiently transfected for 48 hours with 200 ng/dish control pcDNA3.1 plasmid or NTT-MMP-2 pcDNA. Thereafter, H9C2 nuclei were harvested using standard methodology, followed by Western blot for IRF7 using rabbit polyclonal anti-IRF7 antibody (Abcam Ab11980) or rabbit polyclonal anti-IRF1 antibody (Abcam Ab26109). Nuclear extracts from the murine macrophage cell line RAW264.7 (ATCC) were used as a positive control.

## Supporting Information

Figure S1 **Transmission electron microscopy of mitochondrial-enriched fraction from murine hearts demonstrating a high degree of mitochondrial enrichment with minimal membrane contamination.** Membrane fragments are mitochondrial-associated endoplasmic reticulum. (X 15,000).
(TIF)

Figure S2 **Western blot of mitochondrial fractions from control (CON) and fat-fed (ATHERO) ApoER61^h/h^/SF-B1 KO mice.** (rMMP2: recombinant 68 kDa MMP-2 protein).
(TIF)

Figure S3 **PCR-validation of microarray findings.** Data displayed represent means of triplicate determinations and expressed as fold-change as compared to controls.
(TIF)

Table S1 **Microarray transcripts and ontologies up-regulated by NTT-MMP-2.**
(DOCX)

Table S2 **Microarray transcripts and ontologies down-regulated by NTT-MMP-2.**
(DOCX)

Table S3 **Microarray transcripts and ontologies up-regulated (A) and down-regulated (B) by full length MMP-2.**
(DOCX)
