# Supplementary figures and images for: A Novel Intracellular Isoform of Matrix Metalloproteinase-2 Induced by Oxidative Stress Activates Innate Immunity
Source: PLoS One. 2012 Apr 3;7(4):e34177. doi: 10.1371/journal.pone.0034177 (PMC3317925; doi:10.1371/journal.pone.0034177)

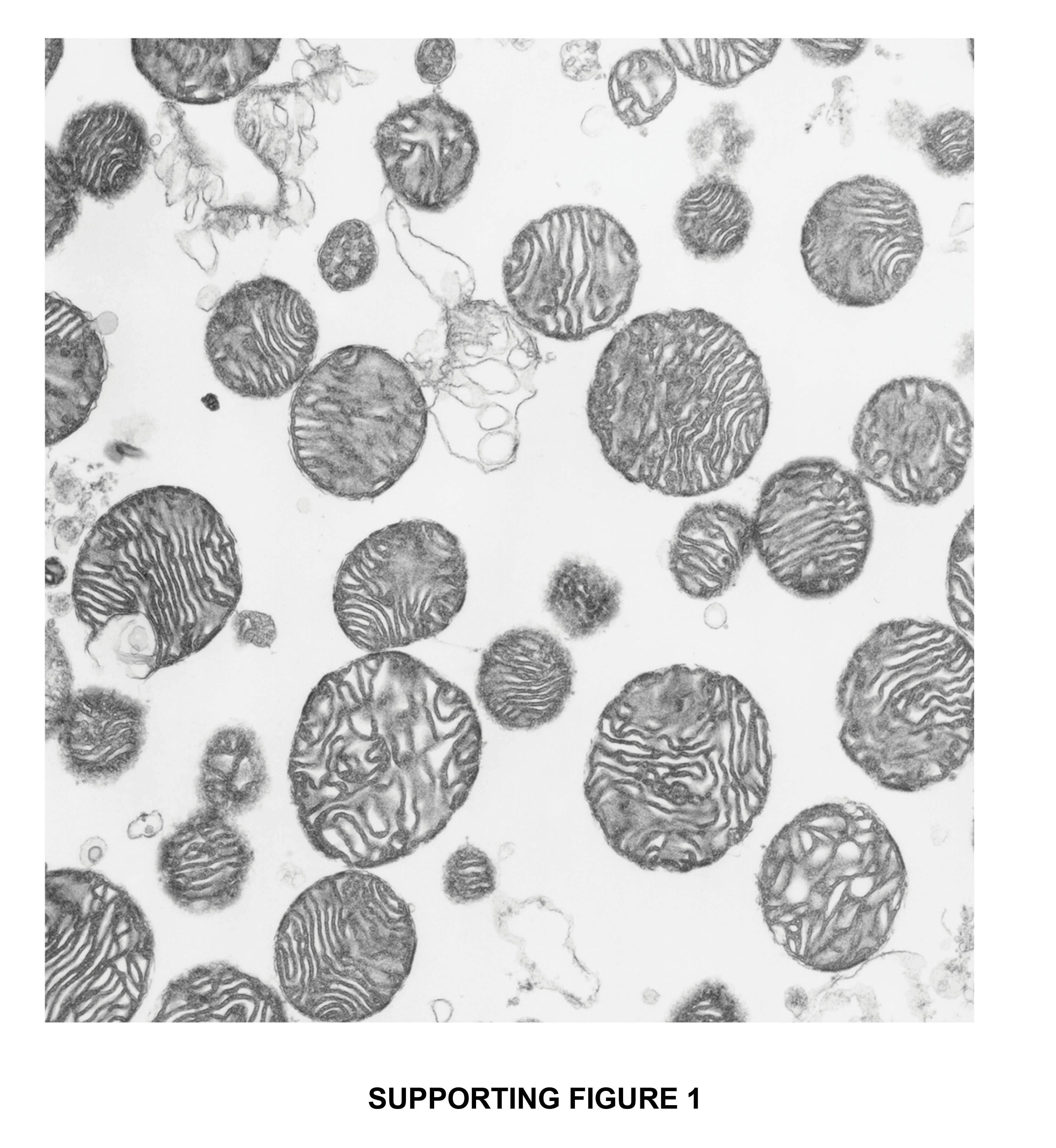

Supplement: Figure S1 — Transmission electron microscopy of mitochondrial-enriched fraction from murine hearts demonstrating a high degree of mitochondrial enrichment with minimal membrane contamination. Membrane fragments are mitochondrial-associated endoplasmic reticulum. (X 15,000). (TIF) [file pone.0034177.s001.tif]

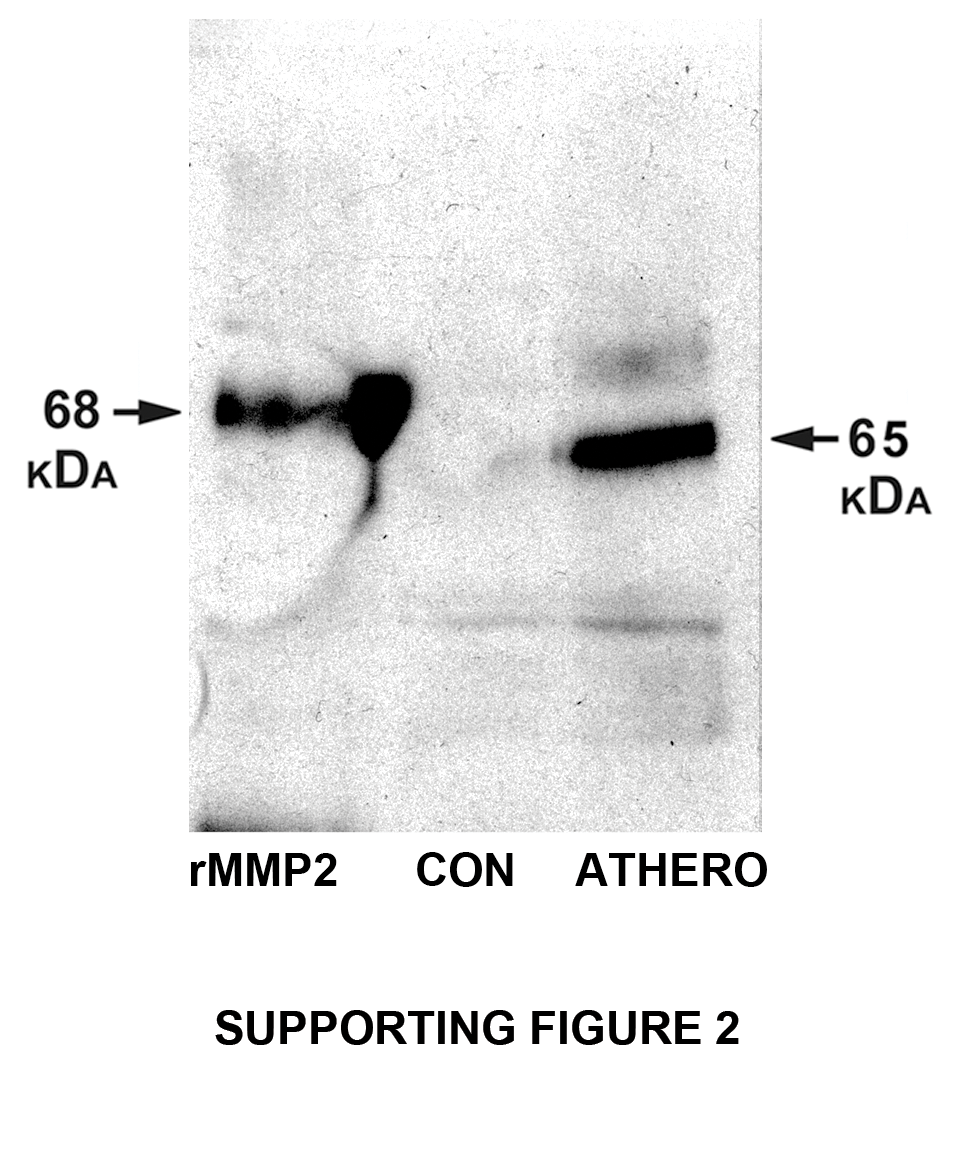

Supplement: Figure S2 — Western blot of mitochondrial fractions from control (CON) and fat-fed (ATHERO) ApoER61h/h/SF-B1 KO mice. (rMMP2: recombinant 68 kDa MMP-2 protein). (TIF) [file pone.0034177.s002.tif]

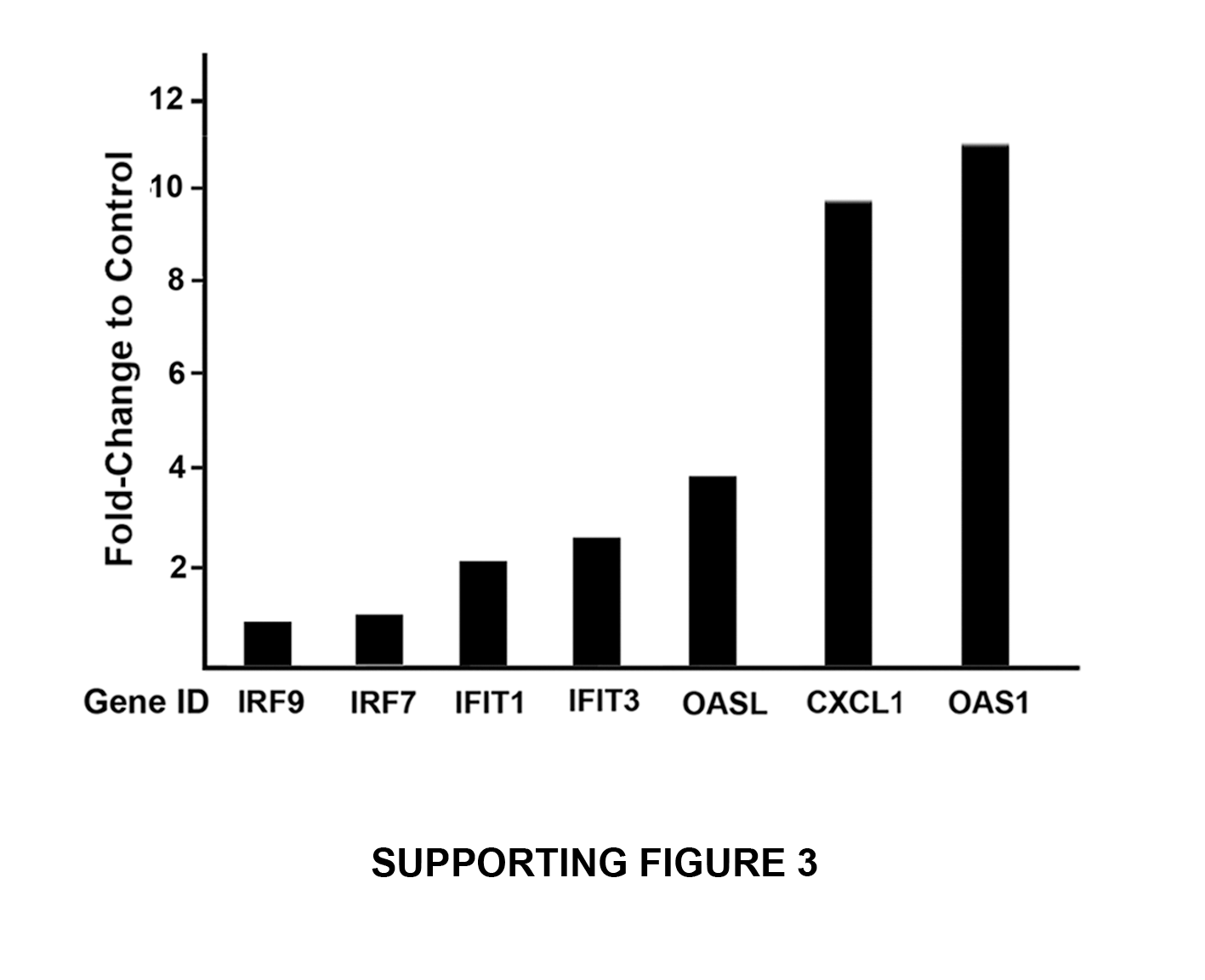

Supplement: Figure S3 — PCR-validation of microarray findings. Data displayed represent means of triplicate determinations and expressed as fold-change as compared to controls. (TIF) [file pone.0034177.s003.tif]
