# Supplementary material for: A Novel Intracellular Isoform of Matrix Metalloproteinase-2 Induced by Oxidative Stress Activates Innate Immunity
Source: PLoS One. 2012 Apr 3;7(4):e34177. doi: 10.1371/journal.pone.0034177 (PMC3317925; doi:10.1371/journal.pone.0034177)
Supplement: Table S1 — Microarray transcripts and ontologies up-regulated by NTT-MMP-2. (DOCX) [file pone.0034177.s004.docx]

| **Table 1: Genes Up-Regulated by NTT-MMP2** | | |
| --- | --- | --- |
| Gene Symbol | Fold-Change | Gene Name |
| *Viral Stress Induced Genes* | | |
| OAS1A | 13.2 | 2’-5’ oligoadenylate synthetase 1 A |
| OAS1B | 10.9 | 2’-5’ oligoadenylate synthetase 1B |
| OASL1 | 9.6 | 2’-5’ oligoadenylate synthetase-like 1 |
| ADAR | 3.0 | Adenosine deaminase-RNA specific |
| IFIT1 | 11.8 | Interferon-induced protein with tetratricopeptide repeats 1 |
| IFIT2 | 8.0 | Interferon-induced protein with tetratricopeptide repeats 2 |
| IFIT3 | 2.1 | Interferon-induced protein with tetratricopeptide repeats 3 |
| PRKRA | 2.1 | Protein kinase, interferon-inducible double stranded RNA-dependent |
| GSTA3 | 4.6 | Glutathione S-transferase 3 |
| GBP2 | 2.1 | Guanylate binding protein 2, interferon-inducible |
| *Viral Stress Induced Transcription Factors* | | |
| IRF7 | 4.0 | Interferon regulatory factor 7 |
| STAT2 | 2.0 | Signal transduction and activator of transcription 2 |
| IFP35 | 2.9 | Interferon-induced protein 35 |
| PARP14 | 3.7 | Poly [ADP-ribose] polymerase 14 |

| Gene Symbol | Fold-Change | Gene Name |
| --- | --- | --- |
| *Ubiquination/Proteosome/Autophagy* | | |
| USP18 | 13.8 | Ubiquitin-specific peptidase 18 ortholog |
| DTX32 | 3.1 | Deltex3-like |
| Ube216 | 3.0 | Ubiquitin-conjugating enzyme E2L 6 ortholog |
| PSMB9 | 2.7 | Proteosome subunit B type 9 |
| IRGM1 | 3.5 | Immunity-related GTPase family M |
| *Chemokines/Cytokines* | | |
| CXCL10 | 5.1 | Chemokine (C-X-C) ligand 10 |
| CCL2 | 3.1 | Chemokine (C-C motif ligand 2/monocyte chemotactic protein-1 |
| IL6 | 2.9 | Interleukin 6 |
| CXCL1 | 2.4 | Chemokine (C-X-C) ligand 1 |
| *Signaling* | | |
| KCNT1 | 2.7 | Potassium channel, subfamily T, member 1 (NA+ activated K channel) |
| ADAP12 | 2.0 | A kinase (PRKA) anchor protein 12 |
| *Other* | | |
| MTA1 | 2.1 | Metallothionein 1A |
| COXA2 | 2.0 | Cytochrome C oxidase subunit VIa, polypeptide 2 (heart) |
