# Supplementary material for: A Novel Intracellular Isoform of Matrix Metalloproteinase-2 Induced by Oxidative Stress Activates Innate Immunity
Source: PLoS One. 2012 Apr 3;7(4):e34177. doi: 10.1371/journal.pone.0034177 (PMC3317925; doi:10.1371/journal.pone.0034177)
Supplement: Table S2 — Microarray transcripts and ontologies down-regulated by NTT-MMP-2. (DOCX) [file pone.0034177.s005.docx]

| **Table 2: Genes Down-Regulated by NTT-MMP2** | | |
| --- | --- | --- |
| Gene Symbol | Fold-Change | Gene Name |
| *Resistance to Apoptosis /Oxidative Stress* | | |
| BCLX | 4.9 | Bcl-like 1 |
| YME1L1 | 4.1 | YME1-like 1 |
| ATRN | 2.9 | Attractin |
| LDLR | 8.3 | Low density lipoprotein receptor |
| HSPD1 | 2.4 | Heat shock protein 1 (chaperonin) |
| *Contractility* | | |
| MYO1D | 10.4 | Myosin ID |
| MYH3 | 3.3 | Myosin heavy polypeptide 3 |
| MYBPH | 3.0 | Myosin binding protein H |
| MYL2 | 2.7 | Myosin light chain-2 |
| TNNI3 | 2.0 | Troponin I (cardiac) |
| *Signaling* | | |
| SH3GL1 | 3.4 | SH3-domain GRB like-1 |
| HRC | 3.0 | Histidine-rich calcium binding protein |
