# Supplementary material for: A Novel Intracellular Isoform of Matrix Metalloproteinase-2 Induced by Oxidative Stress Activates Innate Immunity
Source: PLoS One. 2012 Apr 3;7(4):e34177. doi: 10.1371/journal.pone.0034177 (PMC3317925; doi:10.1371/journal.pone.0034177)
Supplement: Table S3 — Microarray transcripts and ontologies up-regulated (A) and down-regulated (B) by full length MMP-2. (DOCX) [file pone.0034177.s006.docx]

| **Table 3A: Genes Up-Regulated by Full Length MMP2** | | |
| --- | --- | --- |
| Gene Symbol | Fold-Change | Gene Name |
|  | | |
| CTNNA2 | 5.8 | Catenin alpha 2 |
| GSTA3 | 3.9 | Glutathione S-transferase 3* |
| CPB2 | 2.7 | Carboxypeptidase B2 |
| KRT7 | 2.7 | Keratin 7 |
| ADAP12 | 2.7 | A kinase (PRKQ) anchor protein 12* |
| ETV4 | 2.2 | Ets variant 4 |
| **Table 3B: Genes Down-Regulated by Full Length MMP2** | | |
| FILIP1 | 4.0 | Filamin A interacting protein 1 |
| PRKAR2B | 3.7 | Protein kinase, cAMP dependent regulatory unit, type II beta |
| GOT2 | 2.7 | Glutamate oxaloacetate transaminase* |
| FGFR1 | 2.5 | Fibroblast growth factor receptor |
| MYL2 | 2.5 | Myosin light chain-2* |
| HRC | 2.4 | Histidine-rich calcium binding protein * |
| MYBPH | 2.3 | Myosin binding protein H* |
